# Endoscopic manifestation of intestinal transplant-associated microangiopathy after stem cell transplantation

**DOI:** 10.1186/s12876-024-03221-y

**Published:** 2024-04-22

**Authors:** Masaya Iwamuro, Daisuke Ennishi, Nobuharu Fujii, Ken-ichi Matsuoka, Takehiro Tanaka, Toshihiro Inokuchi, Sakiko Hiraoka, Motoyuki Otsuka

**Affiliations:** 1 Department of Gastroenterology and Hepatology, Okayama University Graduate School of Medicine, Dentistry, and Pharmaceutical Sciences, 2-5- 1 Shikata-cho, Kita-ku, 700-8558 Okayama, Okayama Japan; 2 Department of Hematology and Oncology, Okayama University Graduate School of Medicine, Dentistry, and Pharmaceutical Sciences, 700-8558 Okayama, Japan; 3 Department of Pathology, Okayama University Graduate School of Medicine, Dentistry, and Pharmaceutical Sciences, 700-8558 Okayama, Japan

**Keywords:** Colonoscopy, Esophagogastroduodenoscopy, Graft-versus-host disease, Hematopoietic stem cell transplantation, Intestinal transplant-associated microangiopathy, iTAM

## Abstract

**Background:**

Endoscopic features of intestinal transplant-associated microangiopathy (iTAM) have not been comprehensively investigated. This study aimed to examine the endoscopic characteristics of patients diagnosed with iTAM.

**Methods:**

This retrospective analysis included 14 patients pathologically diagnosed with iTAM after stem cell transplantation for hematolymphoid neoplasms (*n* = 13) or thalassemia (*n* = 1). The sex, age at diagnosis, endoscopic features, and prognosis of each patient were assessed. Serological markers for diagnosing transplant-associated thrombotic microangiopathy were also evaluated.

**Results:**

The mean age at the time of iTAM diagnosis was 40.2 years. Patients diagnosed based on the pathognomonic pathological changes of iTAM presented with diverse symptoms at the times of endoscopic examinations, including diarrhea (*n* = 10), abdominal pain (*n* = 5), nausea (*n* = 4), appetite loss (*n* = 2), bloody stools (*n* = 2), abdominal discomfort (*n* = 1), and vomiting (*n* = 1). At the final follow-up, six patients survived, while eight patients succumbed, with a median time of 100.5 days (range: 52–247) post-diagnosis. Endoscopic manifestations included erythematous mucosa (*n* = 14), erosions (*n* = 13), ulcers (*n* = 9), mucosal edema (*n* = 9), granular mucosa (*n* = 9), and villous atrophy (*n* = 4). Erosions and/or ulcers were primarily observed in the colon (10/14, 71%), followed by the ileum (9/13, 69%), stomach (4/10, 40%), cecum (5/14, 36%), duodenum (3/10, 30%), rectum (4/14, 29%), and esophagus (1/10, 10%). Cytomegalovirus infection (*n* = 4) and graft-versus-host disease (*n* = 2) coexisted within the gastrointestinal tract. Patients had de novo prolonged or progressive thrombocytopenia (6/14, 43%), decreased hemoglobin concentration (4/14, 29%), reduced serum haptoglobin level (3/14, 21%), and a sudden and persistent increase in lactate dehydrogenase level (2/14, 14%). Peripheral blood samples from 12 patients were evaluated for schistocytes, with none exceeding 4%.

**Conclusions:**

This study provides a comprehensive exploration of the endoscopic characteristics of iTAM. Notably, all patients exhibited erythematous mucosa throughout the gastrointestinal tract, accompanied by prevalent manifestations, such as erosions (93%), ulcers (64%), mucosal edema (64%), granular mucosa (64%), and villous atrophy (29%). Because of the low positivity for serological markers of transplant-associated thrombotic microangiopathy in patients with iTAM, endoscopic evaluation and biopsy of these lesions are crucial, even in the absence of these serological features.

## Introduction

Intestinal transplant-associated microangiopathy (iTAM) is a rare but serious complication of stem cell transplantation. This disease is characterized by damage to small blood vessels (microangiopathy) in the intestine, leading to ischemia and subsequent tissue injury [1, 2]. iTAM typically presents with abdominal pain, diarrhea, gastrointestinal bleeding, and signs of organ dysfunction. The exact cause of iTAM is not fully understood; however, it is believed to be multifactorial and involves immunological factors, infections, and the effects of immunosuppressive medications used to prevent graft-versus-host disease (GVHD). The pathophysiology of iTAM involves injury to the endothelial cells lining the blood vessels in the intestine. This can lead to the formation of blood clots within vessels and impaired blood flow, resulting in tissue damage. iTAM is usually diagnosed through a biopsy of the affected intestine in addition to clinical evaluation [3, 4].

Treatment of iTAM aims to address the underlying causes and manage complications. Treatments may involve adjustments to the immunosuppressive regimen, treatment of infections, and supportive measures such as fluid resuscitation and nutritional support [1, 5]. iTAM is a complex condition requiring specialized medical care and close monitoring. The prognosis of individuals with iTAM can vary depending on disease severity and treatment response. Timely diagnosis and intervention are crucial for optimizing the outcomes of patients with iTAM. Meanwhile, in the context of hematopoietic stem cell transplantation, the occurrence of intestinal complications after the procedure encompasses GVHD and cytomegalovirus enteritis as notable examples [6–8]. It is crucial to acknowledge that the treatment approaches for iTAM, GVHD, and cytomegalovirus enteritis differ substantially, underscoring the importance of precise differential diagnoses and expeditious therapeutic interventions to effectively manage these conditions. However, the endoscopic characteristics of iTAM have not been sufficiently explored.

This study aimed to assess the endoscopic features and clinical background of iTAM in patients admitted to our hospital.

## Methods

**Fig. 1:**
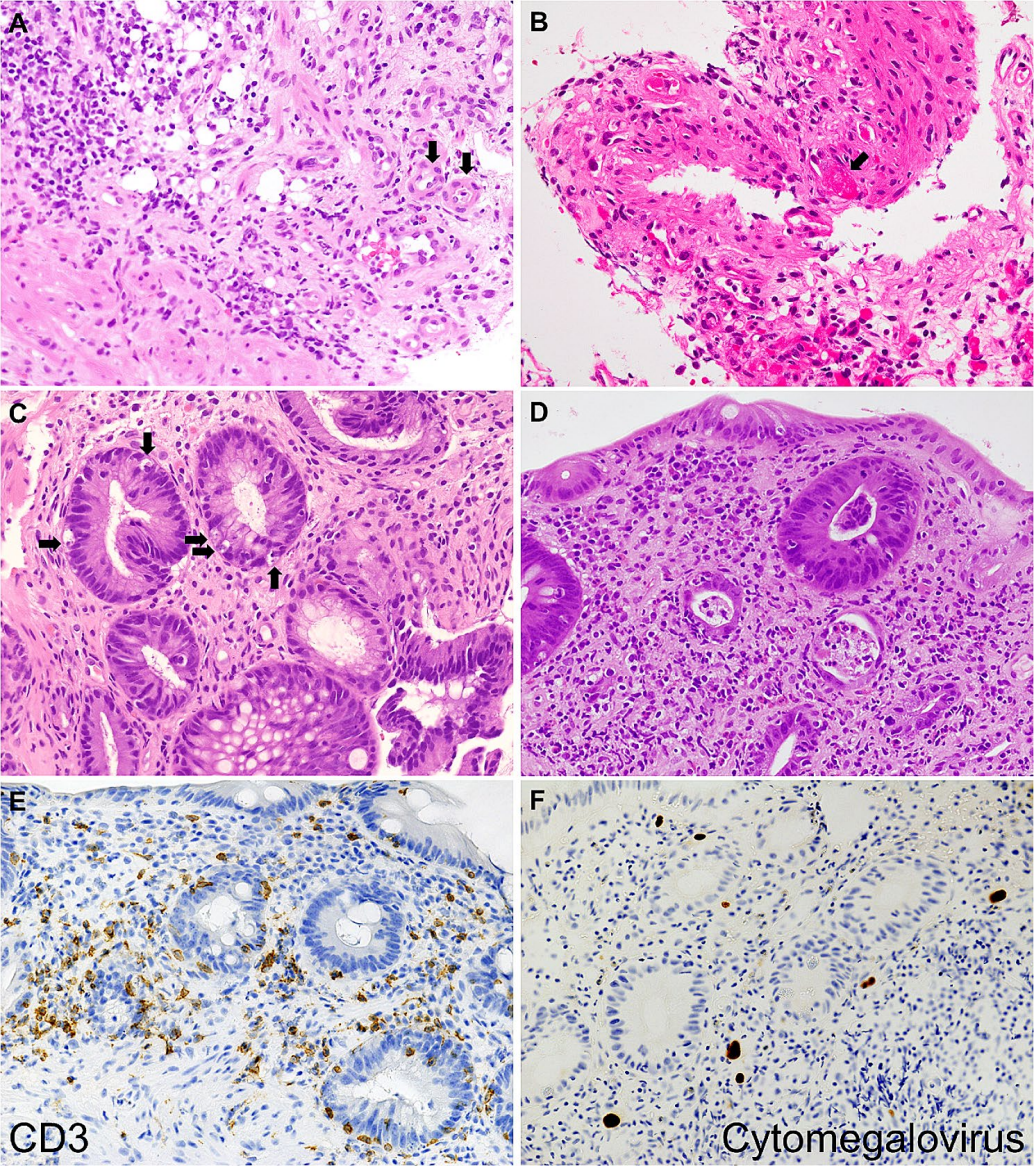
Pathological images. Endoscopic biopsy specimens obtained from the colorectum of a 16-year-old boy exhibit endothelial cell swelling (**A**, arrows). An intraluminal thrombus is focally present (**B**, arrow). The colorectal and ileal mucosa of a 65-year-old woman displays glandular epithelial apoptosis (**C**, arrows), while the presence of inflammatory cell infiltration in the interstitium is sparse. Intestinal GVHD is characterized by extensive, diffuse lymphocyte infiltration within the mucosa (**D**) and the infiltration of CD8 + T lymphocytes into the epithelium (**E**). Gastrointestinal infection with cytomegalovirus was diagnosed based on positive immunostaining for cytomegalovirus (**F**). The pathological images in **D** and **E**, and **F** are presented as representative examples of intestinal GVHD and CMV infection, not the target patients of this study

Patients pathologically diagnosed with iTAM between May 2015 and April 2023 were identified by searching the database of the Department of Pathology at our hospital and were included as the study cohort for this investigation. Two of the patients examined were described in our previous report [9]. Histological diagnoses were made based on endoscopic biopsies. Immunostaining for CD8 and cytomegalovirus was performed on endoscopic biopsy specimens in all patients who underwent stem cell transplantation and were suspected of GVHD, iTAM, or cytomegalovirus enteritis. iTAM was diagnosed based on the presence of any of the following pathological alterations in small blood vessels indicating microangiopathy: endothelial cell swelling, thickening of the blood vessel wall, intraluminal thrombus, vascular hyalinosis, or endothelial cell separation (Fig. 1A and B) [3]. Segmental damage to the gastrointestinal mucosa and the presence of ghost cells (characterized by preserved cellular outlines with empty cell contents) were also considered significant findings for the diagnosis of iTAM. Additionally, epithelial apoptosis of the glands (Fig. 1C), loss of glands, and/or total denudation of the mucosa were observed as tissue injuries subsequent to microangiopathy and ischemia. The diagnosis of GVHD was made based on the extensive lymphocyte infiltration within the mucosa, with diffuse rather than regional changes, and the infiltration of CD8^+^ T lymphocytes into the epithelium along with apoptosis within the crypts (Fig. 1D and E). Gastrointestinal infection with cytomegalovirus was diagnosed based on the positivity of immunostaining for cytomegalovirus (Fig. 1F). We retrospectively examined sex, age at diagnosis, endoscopic and histological features, and prognosis of each patient. Serological markers for diagnosing transplant-associated thrombotic microangiopathy were also evaluated based on the international working group criteria of the European Group for Blood and Marrow Transplantation: schistocytes (fragmented red blood cells) in peripheral blood (> 4%), de novo prolonged or progressive thrombocytopenia (platelet count < 50,000/mm^3^ or 50% reduction from baseline), sudden and persistent increase in lactate dehydrogenase (LDH), decreased hemoglobin concentration, and decreased serum haptoglobin [10].

Association between each endoscopic feature and the outcome (alive or deceased) was assessed with F-tests using JMP 16.0.0 software. This study was approved by the Ethics Committees of Okayama University Hospital (2307-026) and other institutions and adhered to the Declaration of Helsinki. The requirement for written informed consent was waived owing to the observational, non-interventional, and retrospective design of the study. All investigations were performed in accordance with the relevant guidelines and regulations.

## Results

Between May 2015 and April 2023, a total of 348 patients underwent stem cell transplantation in our hospital. During this period, we identified 14 patients (9 men and 5 women) diagnosed with iTAM. Patient characteristics are summarized in Table 1. The mean (± standard deviation [SD]) age at the diagnosis of iTAM was 40.2 ± 19.5 years (range: 11–65 years). The underlying diseases requiring stem cell transplantation included myelodysplastic syndrome (*n* = 6), acute lymphoblastic leukemia (*n* = 3), acute myeloid leukemia (*n* = 1), adult T-cell leukemia/lymphoma (*n* = 1), diffuse large B-cell lymphoma (*n* = 1), thalassemia (*n* = 1), and T-cell lymphoma (*n* = 1). The graft sources for stem cell transplantation were the bone marrow (*n* = 6), peripheral blood (*n* = 5), and cord blood (*n* = 3). Patients who eventually developed iTAM underwent endoscopic examinations for various symptoms such as diarrhea (*n* = 10), abdominal pain (*n* = 5), nausea (*n* = 4), appetite loss (*n* = 2), bloody stools (*n* = 2), abdominal discomfort (*n* = 1), and vomiting (*n* = 1). All patients had received at least one immunosuppressive agent, including corticosteroids (*n* = 13; prednisolone, *n* = 5; hydrocortisone, *n* = 3; methylprednisolone, *n* = 2; prednisolone and methylprednisolone, *n* = 2; prednisolone and dexamethasone, *n* = 1), calcineurin inhibitors (*n* = 12; tacrolimus, *n* = 11; cyclosporin, *n* = 1), immunoglobulin (*n* = 4), anti-thymocyte immunoglobulin (*n* = 1), and/or mycophenolate mofetil (*n* = 1).

**Table 1:** Characteristics of the patients

	Number of patients
Sex	
Male	9
Female	5
Age (years, mean ± SD)	40.2 ± 19.5
Underlying disease	
Myelodysplastic syndrome	6
Acute lymphoblastic leukemia	3
Acute myeloid leukemia	1
Adult T cell leukemia/lymphoma	1
Diffuse large B-cell lymphoma	1
Thalassemia	1
T-cell lymphoma	1
Graft source of stem cell transplantation	
Bone marrow	6
Peripheral blood	5
Cord blood	3
Symptoms	
Diarrhea	10
Abdominal pain	5
Nausea	4
Appetite loss	2
Bloody stools	2
Abdominal discomfort	1
Vomiting	1

SD: standard deviation

**Fig. 2:**
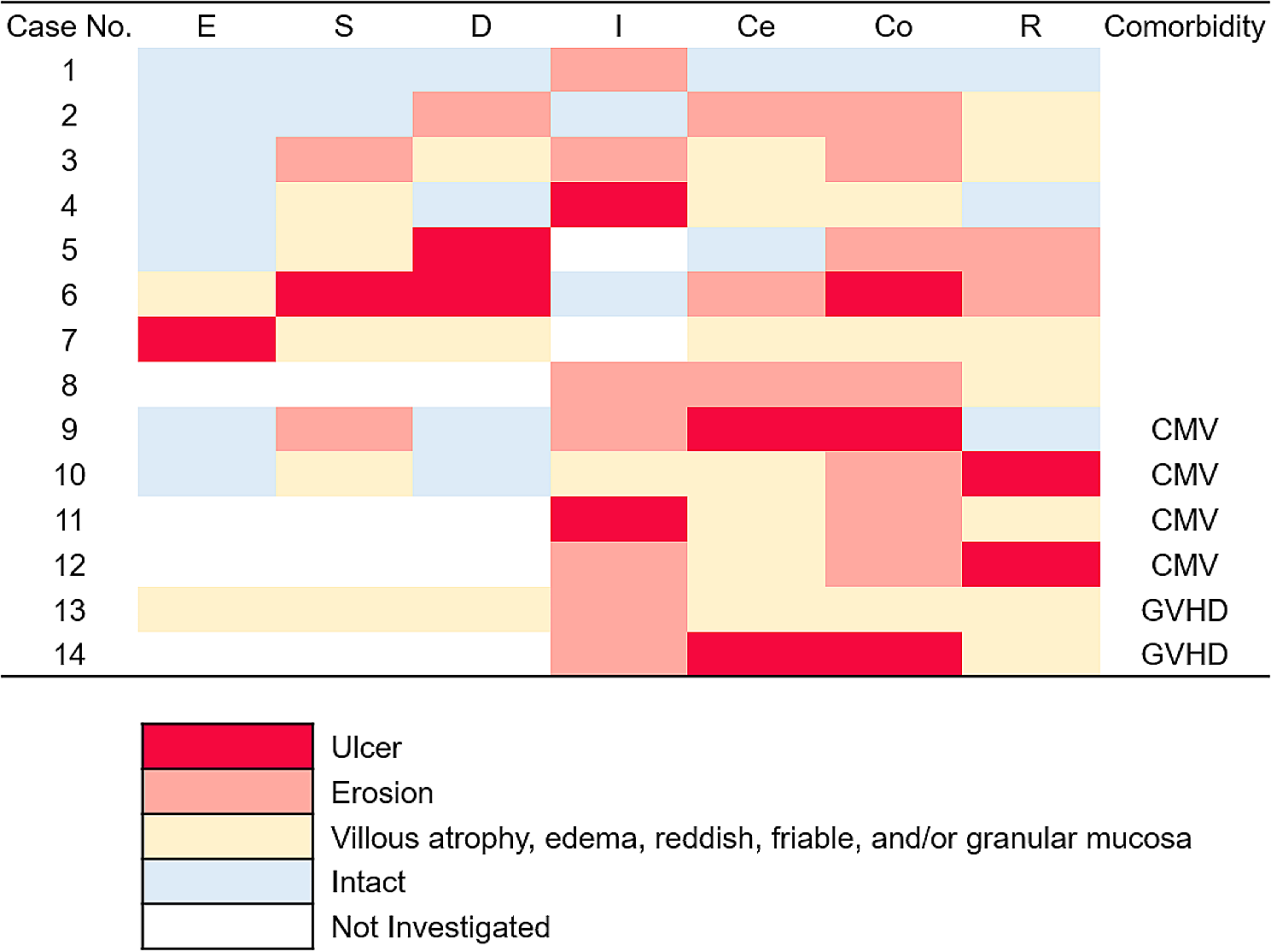
Color charts showing correlation between endoscopic features and affected gastrointestinal segments. E: esophagus; S: stomach; D: duodenum; I: ileum; Ce: cecum; Co: colon; R: rectum; CMV: cytomegalovirus enterocolitis; GVHD: graft-versus-host disease

iTAM was diagnosed at an average of 101 ± 64 days (mean ± SD) following stem cell transplantation, with a range of 21–220 days (Table 2). Endothelial cell swelling/thickening of the blood vessel wall (*n* = 10, 71%) was the most frequently observed on microscopy, followed by intraluminal thrombus (*n* = 4, 29%), vascular hyalinosis (*n* = 4, 29%), and endothelial cell separation (*n* = 2, 14.3%). Segmental damage (*n* = 6, 43%), ghost cells (*n* = 5, 36%), epithelial apoptosis of the glands (*n* = 8, 57%), loss of glands (*n* = 6, 43%), and total denudation of the mucosa (*n* = 6, 43%) were also observed as tissue injuries. At the time of the final follow-up, six patients remained alive, whereas eight patients succumbed at a median time of 100.5 days (range: 52–247 days) following iTAM diagnosis. Endoscopic manifestations included erythematous mucosa (*n* = 14, 100%), erosions (*n* = 13, 93%), ulcers (*n* = 9, 64%), mucosal edema (*n* = 9, 64%), granular mucosa (*n* = 9, 64%), and villous atrophy (*n* = 4, 29%). The correlation between endoscopic features and affected gastrointestinal segments is depicted in Fig. 2. Erosions and/or ulcers were most frequently observed in the colon (10/14, 71%), followed by the ileum (9/13, 69%), stomach (4/10, 40%), cecum (5/14, 36%), duodenum (3/10, 30%), rectum (4/14, 29%), and esophagus (1/10, 10%). Each endoscopic finding (erythematous mucosa, erosions, ulcers, mucosal edema, granular mucosa, and villous atrophy) showed no statistically significant association with the outcome (alive or deceased).

**Table 2:** Endoscopic features and outcome of patients with iTAM

	No. of patients
Diagnosis of iTAM (days after SCT, mean ± SD)	101 ± 64
Endoscopic features	
Erythematous mucosa	14
Erosions	13
Ulcers	9
Mucosal edema	9
Granular mucosa	6
Villous atrophy	4
Eventual outcome	
Alive	6
Dead	8

iTAM: intestinal transplant-associated microangiopathy; SCT: stem cell transplantation; SD: standard deviation

In the present study, cytomegalovirus infection (*n* = 4) or GVHD (*n* = 2) was simultaneously detected in the gastrointestinal tract of six patients with iTAM. Consequently, the presence of cytomegalovirus infections or GVHD may induce mucosal alterations in these patients. Analysis of endoscopic features in patients with iTAM without cytomegalovirus infection or GVHD revealed the presence of erythematous mucosa (8/8, 100%), erosions (7/8, 88%), ulcers (4/8, 50%), mucosal edema (4/8, 50%), granular mucosa (4/8, 50%), and villous atrophy (1/8, 13%).

**Fig. 3:**
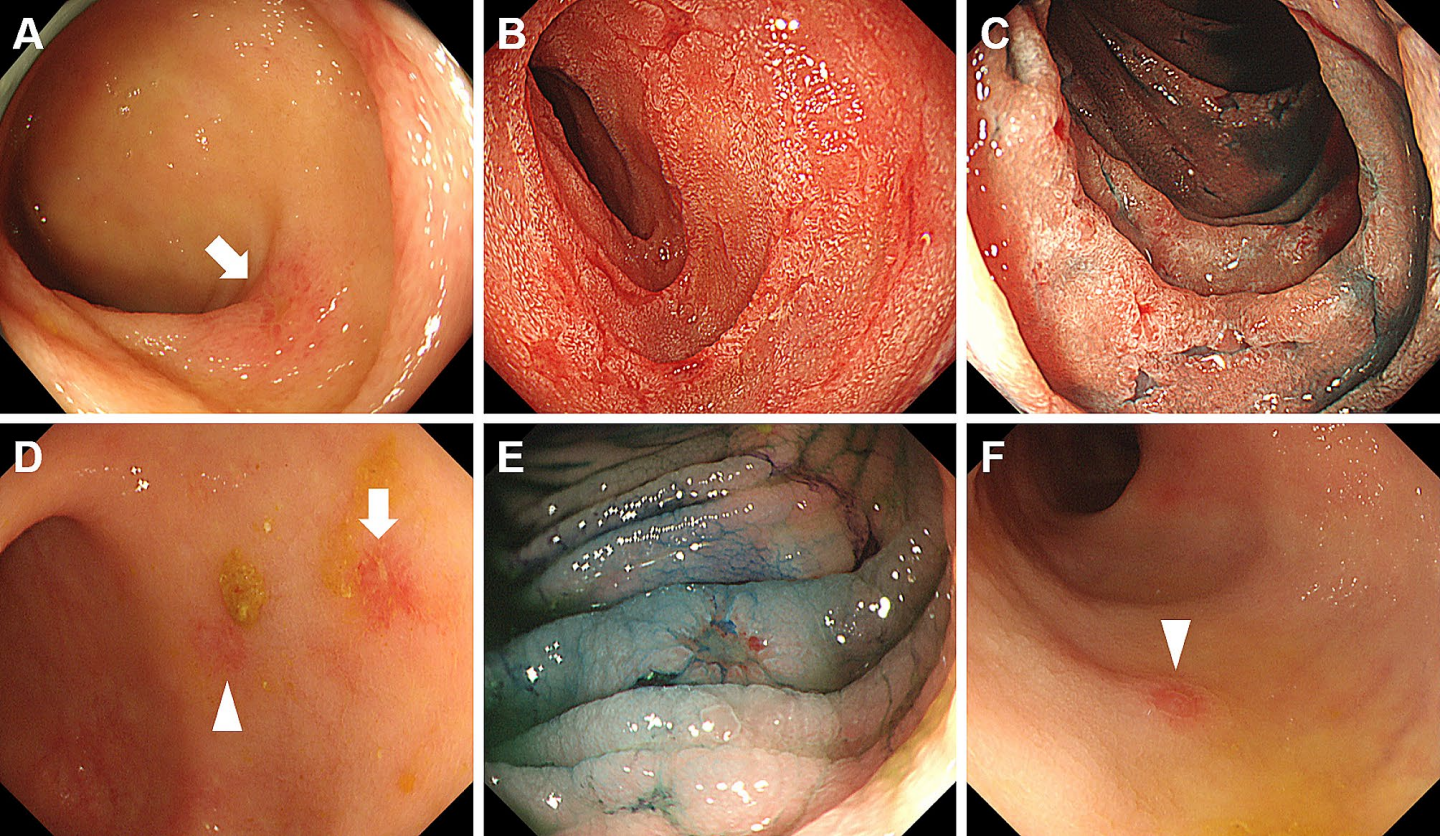
Representative endoscopic images of Cases 1 and 2. A 56-year-old man with intestinal transplant-associated microangiopathy (Case 1), showing erosions observed in the ileum (**A**). A 61-year-old woman (Case 2) demonstrates multiple erosions and villous atrophy in the duodenum (**B** and **C**; C, after indigo carmine spraying). The cecum, colon (**D** and **E**; E, after indigo carmine spraying), and rectum (**F**) display multiple erosions and erythematous mucosa. Indigo carmine spraying highlighted the presence of shallow erosions

**Fig. 4:**
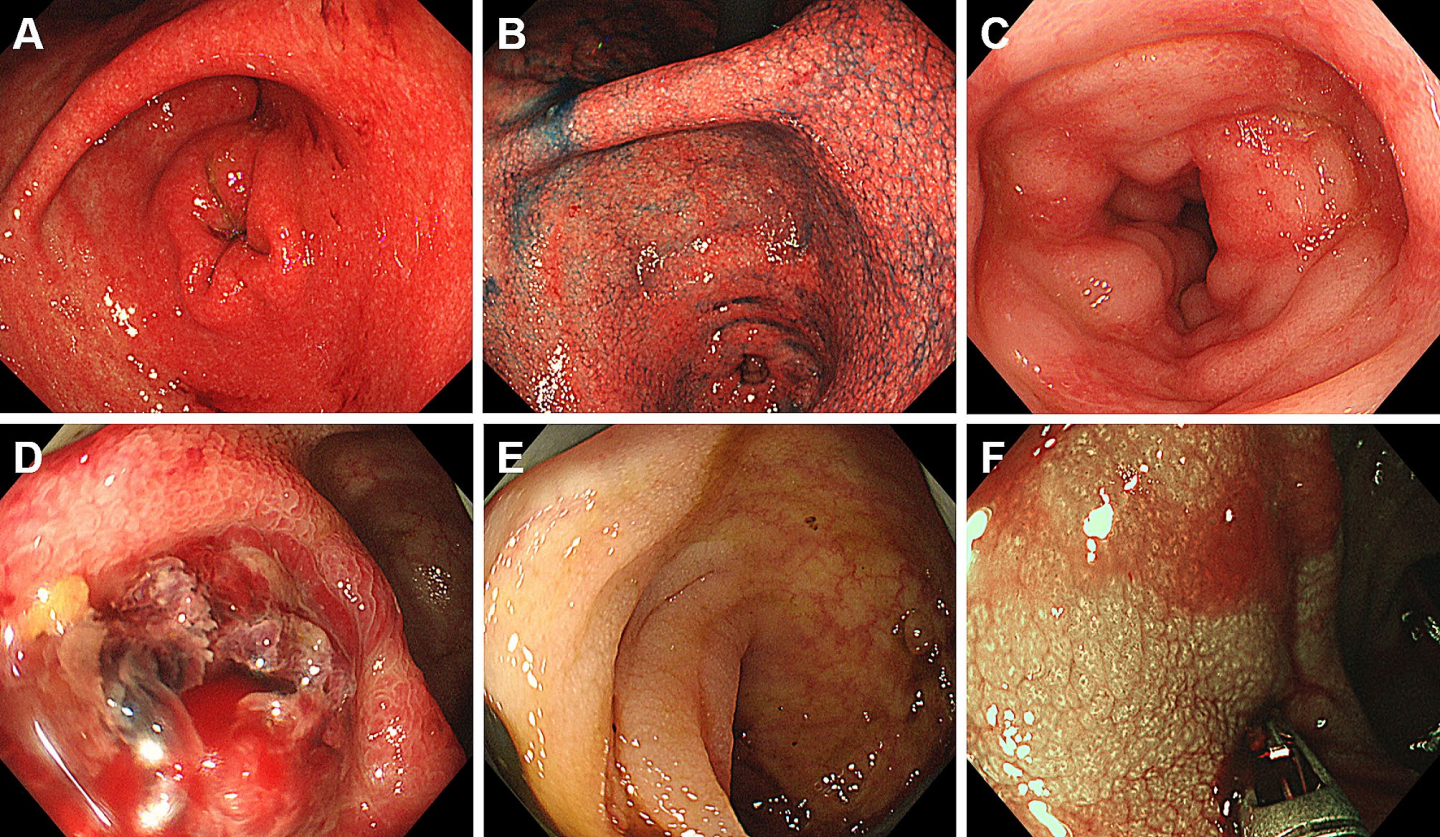
Representative endoscopic images of Cases 3 and 4. Diffuse redness and granular mucosa with erosions are observed in the stomach (**A** and **B**; B, after indigo carmine spraying) of a 16-year-old male patient (Case 3). In the ileum, erythematous and edematous mucosa with erosions is observed (**C**). A hemorrhagic ulcer is visible in the ileum (**D**) of a 58-year-old male patient (Case 4). The vascular pattern is partially indiscernible in the cecum and colon, and the mucosa exhibits a granular appearance (**E** and **F**; F, narrow-band imaging)

**Fig. 5:**
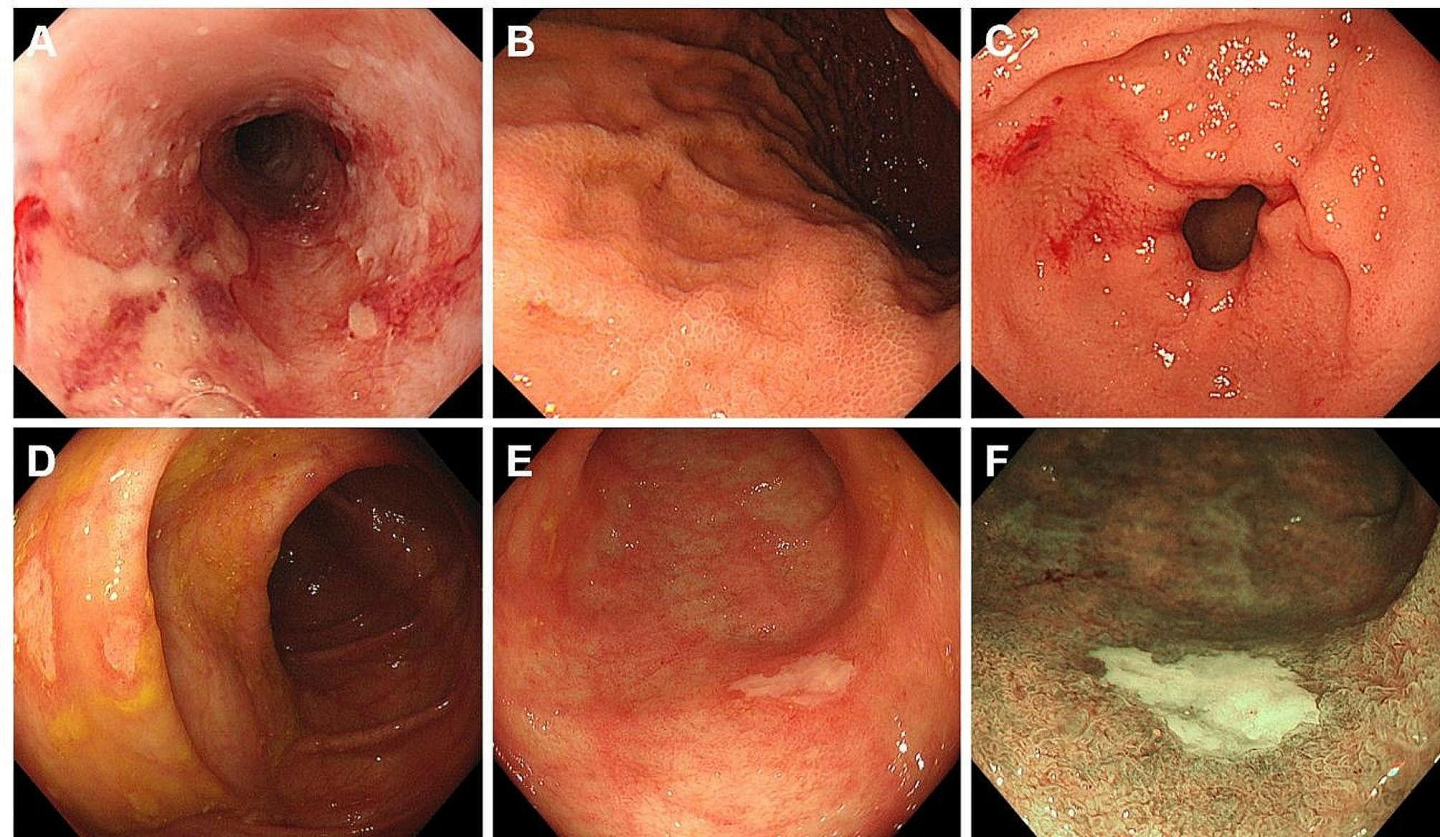
Representative endoscopic images of Cases 7 and 8. A 61-year-old male patient (Case 7) presents with esophageal ulcers (**A**), along with granular mucosa (**B**), erosions, and spontaneous hemorrhages in the stomach (**C**). A 20-year-old female patient (Case 8) displays multiple round erosions in the ileum, cecum, and colon (D–F; F, narrow-band imaging)

Representative endoscopic images of patients with iTAM are shown in Figs. 3, 4 and 5. A 56-year-old man (Case 1) had erosions in the ileum (Fig. 3A) with other parts of the ileum showing erythematous mucosal changes. A 61-year-old woman (Case 2) showed multiple erosions and villous atrophy in the duodenum (Fig. 3B and C; C, after indigo carmine spraying). Multiple erosions and areas of erythematous mucosa were observed in the cecum, colon (Fig. 3D and E; E, after indigo carmine spraying), and rectum (Fig. 3F). Diffuse redness and granular mucosa with erosion were observed in the stomach (Fig. 4A and B; B, after indigo carmine spraying) of a 16-year-old boy (Case 3). Erythematous, edematous mucosa and erosions were observed in the ileum (Fig. 4C). A hemorrhagic ulcer was observed in the ileum (Fig. 4D) of a 58-year-old man (Case 4). The vascular pattern was not partially visible in the cecum or colon, and the mucosa had a granular appearance (Fig. 4E and F; F, narrow-band imaging). A 61-year-old man (Case 7) presented with ulcers in the esophagus (Fig. 5A), granular mucosa (Fig. 5B), erosions, and spontaneous hemorrhages (Fig. 5C) in the stomach. A 20-year-old woman (Case 8) showed multiple round erosions in the ileum, cecum, and colon (Fig. 5D and F; F, narrow-band imaging).

Table 3 shows serological markers for diagnosing transplant-associated thrombotic microangiopathy defined by the international working group criteria [10]. Schistocytes were assessed in the peripheral blood of 12 patients, and none exhibited a schistocyte count exceeding 4%. Enrolled patients most frequently had de novo prolonged or progressive thrombocytopenia defined as platelet count < 50,000/mm^3^ or 50% reduction from baseline level (6/14, 43%), followed by decreased hemoglobin concentration (4/14, 29%), decreased serum haptoglobin (3/14, 21%), and sudden and persistent increase in LDH (2/14, 14%). Eight patients were found positive for one or more serological markers, while the remaining six patients were negative for all markers.

**Table 3:** Serological markers of transplantation-associated thrombotic microangiopathy

	Number of patients
Schistocytes in peripheral blood (> 4%)*	
Present	0
Absent	12
De novo prolonged or progressive thrombocytopenia	
Present	6
Absent	8
Sudden and persistent increase in LDH	
Present	2
Absent	12
Decreased hemoglobin concentration	
Present	4
Absent	10
Decrease in serum haptoglobin	
Present	3
Absent	11

*Not investigated in two patients

LDH: lactate dehydrogenase

## Discussion

In this study, we identified 14 patients with iTAM among a total of 348 patients who underwent stem cell transplantation. Therefore, the incidence rate of iTAM stands at 4.0%, yet likely underestimated compared to the actual incidence rate because some patients have not undergone histological diagnosis of iTAM due to lack of endoscopic examination. We found that all patients with iTAM had erythematous mucosa in the gastrointestinal tract, followed by erosions (93%), ulcers (64%), mucosal edema (64%), granular mucosa (43%), and villous atrophy (29%). In a previous study involving nine patients diagnosed with iTAM through colonoscopic biopsy, all patients were identified as having concomitant GVHD, presenting with edematous and erythematous mucosa exhibiting regions of ulceration and hemorrhage [11]. Another study, which included four patients with concurrent iTAM and GVHD, revealed the presence of erythema, edema, erosion, and ulceration within the gastrointestinal tract [12]. The diagnosis of iTAM primarily depends on the pathological examination of biopsied specimens, focusing on the pathognomonic changes in the small blood vessels outlined above, underscoring the importance of endoscopic biopsy sampling from these lesions and subsequent pathological analysis in patients after stem cell transplantation.

The pathogenesis of iTAM involves microvascular dysfunction, whereas that of GVHD entails an immune reaction to the host. The histological features of iTAM are characterized by ischemic changes in the mucosa associated with microvascular dysfunction. In iTAM, lymphocytic infiltration into the lamina propria is relatively mild, and lymphocytic infiltration into the crypt epithelium is essentially absent, thus characterized by noninflammatory crypt loss [3]. Ischemic injury resulting from endothelial dysfunction leads to wedge-shaped segmental damage to the gastrointestinal mucosa and non-inflammatory apoptosis of cells [3]. In contrast, T lymphocytes play a key role in GVHD. Inflammatory cells are diffusely present within the mucosa, and the immune response induces apoptosis within glandular ducts, which is an essential feature of GVHD.

For the management of acute GVHD, a recommended approach involves the concurrent administration of corticosteroids and calcineurin inhibitors. Immunostimulatory interventions such as anti-thymocyte globulin are pertinent in refractory cases. Although a definitive treatment has not been established for iTAM, the reduction of calcineurin inhibitors has been prioritized [13]. In contrast to the treatment approach used in GVHD, caution should be exercised against the intensification of immunosuppression for iTAM, leading to opposing treatment principles, thus making the differentiation between the two conditions crucial. However, based on the results of our present study, distinguishing between iTAM and intestinal GVHD solely through endoscopic findings seems challenging, despite the presence of pathological differences. In our retrospective analysis of patients with intestinal GVHD, we observed edema, erosion, erythema, a tortoise-shell-like appearance, superficial or deep ulcers, congestion, and villous atrophy during colonoscopic examination [14]. Mucosal abnormalities are frequently identified in patchy or segmented distributions across the gastrointestinal tract. These characteristics have also been delineated in earlier reports by other researchers [15, 16]. Thus, intestinal GVHD and iTAM share, to some extent, similar endoscopic features [17]. Furthermore, it is essential to note that iTAM is often observed in conjunction with GVHD [12]. Further research comparing endoscopic features between iTAM and intestinal GVHD is needed to identify disparities in endoscopic findings, thereby aiding in distinguishing between these two conditions during endoscopy examinations, although it is uncertain whether such differences truly exist.

We also observed that four patients concomitantly exhibited cytomegalovirus infection in the gastrointestinal tract. Cytomegalovirus infection is a significant predisposing factor for the development of transplant-associated thrombotic microangiopathy. Cytomegaloviruses directly invade endothelial cells, eliciting endothelial injury and fostering a prothrombotic milieu [18]. Furthermore, it provokes an inflammatory response, triggers complement activation, and induces endothelial dysfunction, collectively contributing to the pathogenesis of transplant-associated thrombotic microangiopathy. A study conducted by Ye et al. revealed a higher prevalence of cytomegalovirus viremia in 26 patients with transplant-associated thrombotic microangiopathy than in 52 matched controls (42% vs. 12%, *p* < 0.01) [19]. Similarly, an investigation by Ramgopal et al. in pediatric patients demonstrated a greater incidence of cytomegalovirus infection in 93 patients with transplant-associated thrombotic microangiopathy than in 12,369 patients without thrombotic microangiopathy (19.4% vs. 7.7%, *p* < 0.01) [20]. These findings underscore the imperative nature of vigilant monitoring for cytomegalovirus infection or reactivation, as well as consideration of antiviral prophylaxis or preemptive therapy, particularly in high-risk individuals following hematopoietic stem cell transplantation.

The differentiation of iTAM from GVHD and cytomegalovirus infection is crucially important clinically. The international working of the European Group for Blood and Marrow Transplantation attempted to establish diagnostic criteria for the diagnosis of transplant-associated thrombotic microangiopathy. Their criteria included the presence of schistocytes in the peripheral blood (> 4%), persistent or progressive thrombocytopenia (platelet count < 50,000/mm^3^ or a 50% reduction from baseline), a sustained and abrupt rise in LDH levels, decreased hemoglobin concentration, and decreased serum haptoglobin levels. Unfortunately, as shown in Table 3, none of the patients had schistocytes exceeding 4% in their peripheral blood. The prevalence of the other features ranged only from 14 to 43%. Eight patients were found positive for one or more serological markers, while the remaining six patients were negative for all markers. None of the patients met any diagnostic criteria. A prospective study also revealed the presence of iTAM in patients lacking laboratory findings consistent with transplant-associated thrombotic microangiopathy, based on the above diagnostic criteria [17]. Thus, the positivity rates of previously proposed serological markers for transplant-associated thrombotic microangiopathy were not high in patients with iTAM. Consequently, these serological markers are considered not useful for diagnosing iTAM or distinguishing it from intestinal GVHD or cytomegalovirus infection. We consider endoscopic evaluation and biopsy important in patients presenting with gastrointestinal symptoms after hematopoietic stem cell transplantation, even without features of transplant-associated thrombotic microangiopathy.

This study has several limitations that warrant discussion. First, the aim of our study is to introduce iTAM, which has not been thoroughly examined in terms of endoscopic findings thus far. We exclusively reviewed iTAM patients without making any comparisons with patients suffering from GVHD or cytomegalovirus infection. Consequently, specific endoscopic findings unique to iTAM have not been evaluated. To elucidate endoscopic findings for discriminating between iTAM, GVHD, and cytomegalovirus infection, it is imperative to compare and examine patients with these conditions. Another limitation inherent to our study was the relatively modest cohort size, which directly corresponds to the rare occurrence of this disease. Therefore, an extensive exploration of iTAM through a multicenter study featuring an expanded sample size is imperative to elucidate the authentic essence of this infrequent yet perilous condition. Third, although we made the diagnosis of iTAM based on any of the features of microangiopathy, such as endothelial cell swelling, thickening of the blood vessel wall, intraluminal thrombus, vascular hyalinosis, and endothelial cell separation, these pathological findings may also be observed in GVHD and cytomegalovirus enteritis. Thus, underestimation of patients with GVHD and gastrointestinal cytomegalovirus infection, and overestimation of patients with iTAM, might have occurred. Therefore, the pathological diagnosis of iTAM remains a challenge for the future.

In conclusion, we explored the endoscopic characteristics of iTAM. Due to the low prevalence of serological markers associated with transplant-associated thrombotic microangiopathy in patients with iTAM, endoscopic evaluation and biopsy of the gastrointestinal lesions are crucial, even in the absence of such serological features. Additionally, despite the distinct pathologies between iTAM and GVHD, the findings of the current study suggest that both conditions demonstrate similar endoscopic manifestations to a certain degree. While this study exclusively targeted iTAM patients, further investigation including all hematopoietic stem cell transplantation recipients would illuminate endoscopic findings pertinent to discriminating between iTAM, GVHD, and even cytomegalovirus infection.

## Data Availability

The data that support the findings of this study are available from the corresponding author (MI) on reasonable request. The data are not publicly available due to their containing information that could compromise the privacy of research participants.
